# Safety assessment for ibuprofen using disproportionality analysis: a decade of data from the Romanian national pharmacovigilance system

**DOI:** 10.3389/fphar.2026.1863824

**Published:** 2026-08-12

**Authors:** Cristina Anamaria Buciuman, Anca Butuca, Adina Frum, Felicia Gabriela Gligor, Mariana Ganea, Laura Grațiela Vicaş, Eleonora Marian, Octavia Gligor, Carmen Maximiliana Dobrea, Corina Florentina Moisa, Annamaria Pallag, Tunde Jurca, Claudiu Morgovan

**Affiliations:** 1 Inspection Department, National Agency for Medicines and Medical Devices of Romania, Bucharest, Romania; 2 Preclinical Department, Faculty of Medicine, “Lucian Blaga” University of Sibiu, Sibiu, Romania; 3 Department of Pharmacy, Faculty of Medicine and Pharmacy, University of Oradea, Oradea, Romania; 4 Department of Preclinical Disciplines, Faculty of Medicine and Pharmacy, University of Oradea, Oradea, Romania

**Keywords:** adverse drug reactions, anti-inflammatory, disproportionality analysis, drug safety profile, ibuprofen, pharmacovigilance, Romania

## Abstract

**Introduction:**

The aim of this study was to evaluate the adverse reactions associated with ibuprofen, one of the most widely used analgesic-antipyretic agents, in comparison with other nonsteroidal anti-inflammatory drugs (NSAIDs). In Romania, most pharmaceutical forms of ibuprofen are available over the counter, except for parenteral formulations and high-dose oral forms (800 mg), which require a prescription.

**Methods:**

Descriptive and disproportionality methods were conducted on reports associated with ibuprofen and six other NSAIDs, submitted to the National Agency for Medicines and Medical Devices of Romania between 2015 and 2024 by healthcare professionals and patients. The type and the frequency of adverse reactions, and their classification according to the System Organ Classification were analyzed. For the disproportionality analysis, the reporting odds ratio and 95% confidence interval were used to compare the disproportional reporting ADRs related to ibuprofen with that of other six NSAIDs.

**Results:**

From the total number of reports (n = 128), 44 were related to ibuprofen, with an annual mean of 4.4 ± 2.91, indicating greater reporting variability. Within 15 reports, several serious events were recorded 10 required hospitalization or prolongation of hospitalization, 5 were life-threatening, 3 were associated with other severe medical conditions, and one resulted in the patients’ death. The safety profile of ibuprofen shows a predominance of gastrointestinal adverse reactions (n = 31) over immunological (n = 18), cutaneous (n = 18), or neurological reactions (n = 7). However, the disproportionality analysis, limited by the relatively small number of reports, suggests a higher disproportional reporting of gastrointestinal disorders for ibuprofen than for etoricoxib (ROR 4.74; 95% CI 1.72–13.03). In contrast, a lower disproportional reporting skin and soft tissue ADRs related to ibuprofen, compared with diclofenac (ROR 0.32; 95% CI 0.11–0.95) and celecoxib (ROR 0.17; 95% CI 0.05–0.54), was observed.

**Conclusion:**

The reported adverse reactions were consistent with the established safety profile of ibuprofen and are largely described in the Summary of Product Characteristics, with no new safety signals identified. However, this study adds national real-world pharmacovigilance evidence by demonstrating a higher disproportional reporting of gastrointestinal disorders compared with etoricoxib and by highlighting children and adolescents as the most frequently represented reporting population. These findings contribute to a better understanding of ibuprofen safety patterns in routine clinical practice in Romania. The results should be interpreted in the context of inherent limitations, such as underreporting of adverse reactions and the difficulty in establishing causality, particularly for patient-reported cases, which may introduce a higher degree of subjectivity.

## 1 Introduction

Non-steroidal anti-inflammatory drugs (NSAIDs) are used for their analgesic, antipyretic, and anti-inflammatory properties (Kaduševičius, 2021; Panchal and Prince, 2023). Their mechanism of action is based on the inhibition of the cyclooxygenase (COX) enzyme’s activity, which prevents the conversion of arachidonic acid into prostaglandins–mediators of the inflammatory processes (Panchal and Prince, 2023). Three isoforms of the COX enzyme have been identified, but only the first two are known to be responsible for physiological processes in the human body and play an important role in pain and inflammation management (Bokhtia et al., 2023). COX-1 is a constitutive enzyme which is present in most tissues and it is responsible for maintaining homeostasis through functions such as protecting the gastric mucosa, regulating renal blood flow and facilitating platelet aggregation and COX-2 is an inducible isoform whose expression is upregulated by inflammatory stimuli and cytokines, which act as the primary drivers of prostaglandin production at the tissue injury site (Kaduševičius, 2021; Panchal and Prince, 2023; Sohail et al., 2023).

The classification of NSAIDs is consistent with the degree of selectivity for COX isoforms: selective COX-1 inhibitors (low-dose aspirin), non-selective inhibitors (ibuprofen, diclofenac, naproxen), preferential COX-2 inhibitors (meloxicam, nimesulide), and selective COX-2 inhibitors, known as coxibs (celecoxib, etoricoxib) (Panchal and Prince, 2023; Doomra and Goyal, 2020).

In Romania these medications are available in various pharmaceutical forms, including tablets, capsules, suspensions, granules for oral solution, suppositories, topical formulations (i.e., gels, creams, foams, sprays), and injectable or infusible solutions. They are dispensed either by using a medical prescription or as over the counter (OTC) products, thus they are frequently used as self-medication to alleviate pain or fever (Lista Medicamentelor Din Nomenclator, 2026). Due to its high accessibility and the fact that many pharmaceutical forms are available as OTC medication, ibuprofen is one of the most used NSAID in Romania to alleviate pain, fever and inflammation (Lista Medicamentelor Din Nomenclator, 2026; Beldean-Galea et al., 2025).

Despite their therapeutic benefits, NSAIDs present risks and limitations that demand much consideration. Their use is associated with several adverse reactions (Panchal and Prince, 2023; Varrassi et al., 2020; Buciuman et al., 2026) like gastrointestinal disorders (i.e., diarrhoea, abdominal pain, dyspepsia) (Sohail et al., 2023; Hijos-Mallada et al., 2022; Wirth et al., 2024), cardiovascular events (i.e., hypertension, stroke) (Wirth et al., 2024; Schjerning et al., 2020; Ozen et al., 2023), renal impairment (LaForge et al., 2023; Xu et al., 2024; Bensman, 2020), immune system disorders (i.e., angioedema, urticaria), skin disorders (Tjenggal et al., 2025; Melikoglu et al., 2022) or nervous system disorders (Panchal and Prince, 2023; Buciuman et al., 2025a). These adverse reactions depend on the exact medication administered and patients’ interindividual variability (Wirth et al., 2024). Consequently, adverse reaction monitoring is necessary, especially among special classes of consumers, like pregnant women, children or older patients who often administer polymedication, thus increasing the risk of drug interactions (Buciuman et al., 2025b; Sienkiewicz et al., 2021; Cavkaytar and Arga, 2022).

To reduce these clinical risks and ensure patient safety, pharmacovigilance systems provide the tools for monitoring and identifying adverse events (ANMDMR, 2026). The European Union has a thorough system in place to plan for drug safety, monitor medicines after they are approved, and take action to protect public health. This system functions through the coordination of national regulatory authorities across Member States, the European Commission, and the European Medicines Agency (European database of suspected adverse, 2026).

The National Agency for Medicines and Medical Devices of Romania (NAMMDR) is the Romanian national competent authority in the field of medicinal products for human use and medical devices. This institution also holds the central role of monitoring the safety of medicines post-authorization to detect, assess, understand, and prevent adverse reactions or any other problems related to their use in Romania (ANMDMR, 2026).

Due to the high usage of NSAIDs for their analgesic and antipyretic properties, the need to assess their safety arises, thus this study aims to conduct a retrospective analysis of spontaneous adverse drug reactions reported in Romania for ibuprofen–a highly used drug–by comparing its safety profile to other NSAIDs in order to increase clinical awareness for healthcare providers and general population regarding the usage of the studied drugs and thus promote drug safety monitoring and reporting.

## 2 Materials and methods

### 2.1 Study design

Individual case safety reports (ICSRs) associated with ibuprofen use in Romania were used for this study. The reports were submitted to the National Agency for Medicines and Medical Devices of Romania (NAMMDR) by healthcare professionals (physicians and pharmacists) and patients. The primary unit of analysis was the ICSR (case/report). Each ICSR was defined as a single report and could contain one or more suspected adverse drug reactions (ADRs). ADRs were coded according to the Medical Dictionary for Regulatory Activities (MedDRA) terminology and were analyzed at the level of Preferred Terms (PTs) and System Organ Classes (SOCs). Descriptive analyses were performed at both report-level (ICSRs) and event-level (PTs/SOCs), depending on the variable of interest.

We evaluated the reports recorded for ibuprofen, between 2015–2024, compared to other NSAIDs (celecoxib, etoricoxib, diclofenac, nimesulide, ketoprofen, naproxen). The study consisted of the descriptive analysis of the case reports and their descriptive statistics. To begin with, the evolution of reports during this period was analyzed for ibuprofen compared to the NSAIDs taken in the study. Furthermore, the reports were analyzed in terms of demographic characteristics (gender, age). The reports were classified according to the age of the patient in one of the general categories: (<2 months, 2 months–2 years, 3–11 years, 12–17 years, 18–64 years, 65–84 years, ≥85 years, unspecified). Another important aspect that was analyzed was the severity of the cases, the distribution of serious/non-serious cases being carried out. Seriousness at the individual case safety report (ICSR) level was assessed using standard pharmacovigilance criteria (death, life-threatening condition, hospitalization or prolongation of hospitalization, disability or permanent damage, congenital anomaly/birth defect, and other medically important conditions). Multiple seriousness criteria could be assigned to a single ICSR; therefore, seriousness categories were not mutually exclusive, and the cumulative number of seriousness criteria may exceed the number of serious ICSRs (Butuca et al., 2024).

Also, the ratio of ADRs/report for each drug included in the study was calculated, and a comparison between NSAIDs was also made from this point of view.

Subsequently, a stratified analysis of the reports was carried out that included ibuprofen from point of view of the outcome, detailing serious cases (life-threatening cases, cases requiring hospitalization or prolongation of hospitalization, other serious situations, death), as well as the analysis of the drugs co-administered with ibuprofen (Help to shape the MedDRA, 2026).

Another aspect followed was the analysis of the adverse drug reactions reported for ibuprofen. After the identification of ADRs, they were grouped into SOCs, according to the Medical Dictionary for Regulatory Activities (MedDRA) (Help to shape the MedDRA, 2026).

Subsequently, the distribution of the ADRs in the SOCs was performed, the analysis of the PTs in each SOC and the disproportionality analysis compared to the rest of the NSAIDs.

### 2.2 Data extraction and data handling

The data extraction was performed on 1–31 August 2025. Duplicate reports were identified and removed during data cleaning. In cases of follow-up reports, information was merged with the initial ICSR to avoid duplication. Drug names were standardized according to the International Nonproprietary Name (INN) system. Inclusion criteria were defined based on two parameters: the presence of the study drugs of interest and the reporting period. All reports submitted between 2015 and 2024 that listed at least one of the following NSAIDs as suspected drug: ibuprofen, celecoxib, etoricoxib, diclofenac, nimesulide, ketoprofen, or naproxen were included in the analysis. Only drugs reported as suspected were included in the analysis; concomitant medications were recorded but not considered suspected drugs unless explicitly stated. All available pharmaceutical formulations (oral, topical, and parenteral) were included in the analysis and pooled under the respective active substance. Missing data for individual variables (e.g., age, outcome, or co-medication) were coded as “unspecified” and included in the denominators where applicable. Marketing Authorization Holder (MAH) reports submitted to EudraVigilance were not included in the dataset to ensure consistency of reporting structure and homogeneity of the analyzed data source. Reports involving other medications or submitted outside the specified time frame were excluded.

### 2.3 Statistical analysis

#### 2.3.1 Descriptive statistical analysis of cases

The data collected on reported cases for the ibuprofen and other six NSAIDs (celecoxib, etoricoxib, diclofenac, nimesulide, ketoprofen, and naproxen) were subjected to descriptive statistical analysis to assess the distribution of reports over the study period. The following were assessed: (i) Central tendency (arithmetic mean, median and modulus); (ii) Dispersion and variability (standard deviation (SD), standard error (SE), variance, range and interquartile range (IQR) to assess the degree of dispersion of data around the mean); (iii) the form of distribution: skewness and skewness (Kurtosis) used to assess the normality of the distribution of data for each medicinal product.

#### 2.3.2 Disproportionality analysis

In order to identify differences in reporting ibuprofen compared to other six NSAIDs, a disproportionality analysis was performed. A high disproportional reporting indicates a positive association between the drug analyzed and the reported ADR. According to EV, for a disproportionate reporting, two conditions must be fulfilled: (i) the number of ICSRs was at least 5, and (ii) 95% CI of the reporting odds ratio (ROR) was greater than 1.0 (MedCalc’s, 2026).

Because in some instances the number of reports in one of the cells of the 2 × 2 contingency table was zero, resulting in undefined ROR estimates due to division by zero, a Haldane–Anscombe continuity correction of 0.5 was applied (Noguchi et al., 2021). Specifically, 0.5 was added to each cell of the 2 × 2 contingency table.

All descriptive and statistical analyses were performed using Microsoft Excel for Microsoft 365 MSO (Version 2503) and the ROR calculation and 95% confidence interval using MedCalc Software Ltd. *Odds Ratio Calculator*, Version 22.001 (MedCalc’s, 2026).

#### 2.3.3 Ethics

The aggregated data analyzsed does not contain personal information through which patients can be identified, which is why the agreement of the ethics committees is not required.

## 3 Results

### 3.1 Comparative analysis of reports related to ibuprofen versus other NSAIDs

**FIGURE 1:**
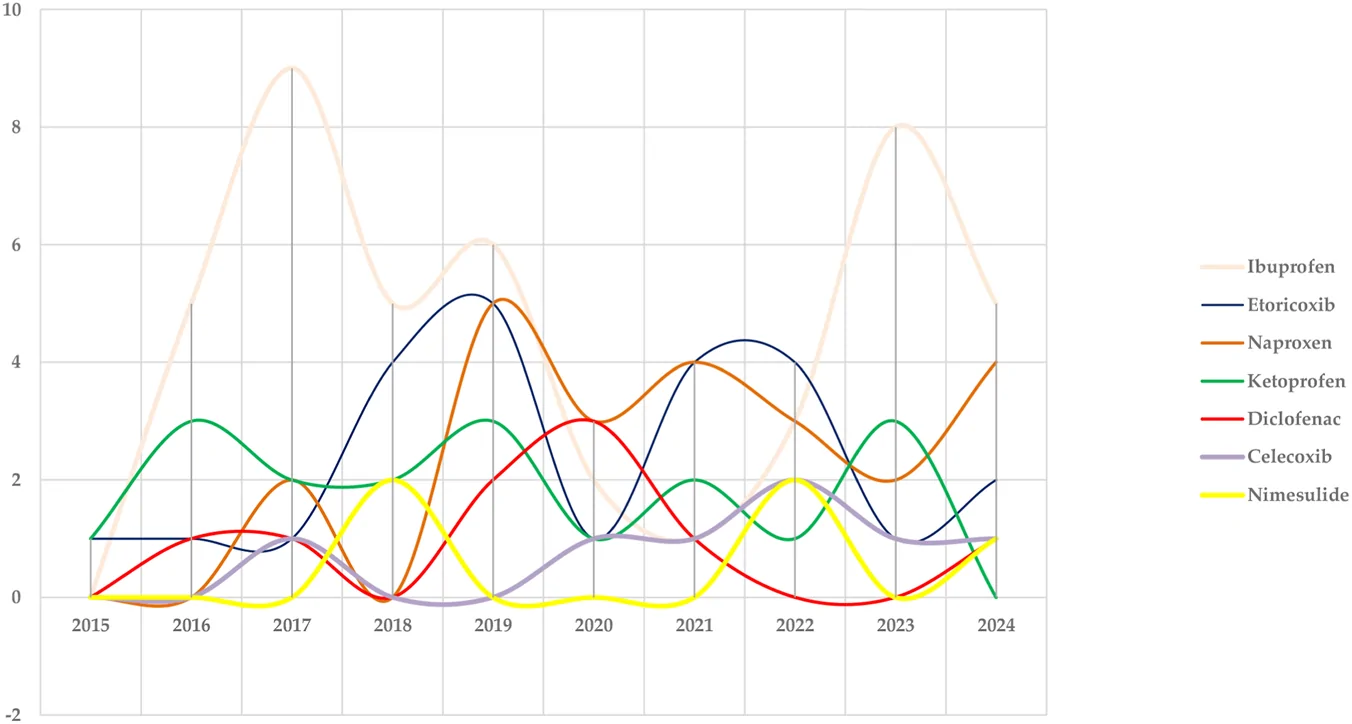
The evolution of reports for NSAIDs between 2015–2024 (n = 128).

According to Figure 1, the peak reporting year for all NSAIDs analyzed was 2019 (n = 21). Although etoricoxib had a lower total number of reports overall (n = 24), it was the only drug with ADR reports submitted every year. Also, relatively high number of reports compared to the other NSAIDs were observed for naproxen (n = 23) and ketoprofen (n = 18).

The highest number of reports was noticed for ibuprofen over the 10-year period (n = 44). From the data submitted to NAMMDR, it can be seen that there is a fluctuating trend (Figure 1), due mostly to the sharp decrease in reports during the COVID-19 pandemic. For example, in 2020 there were the fewest reports (n = 10) since 2016, for all 7 NSAIDs. On the other hand, for ibuprofen in the period 2020–2022 only 6 reports were recorded.

Moreover, the highest mean yearly number of reports was observed for ibuprofen (4.4 ± 2.91), indicating a greater reporting variability. In fact, the high standard deviation (SD) for ibuprofen (SD 2.91) and etoricoxib (SD 1.65) indicates year-to-year variation in report counts. Moreover, a high interquartile range (ibuprofen IQR 3.50, naproxen IQR 3.25, and etoricoxib IQR 3.00) indicates a wider dispersion of the central 50% of yearly values, with alternating years with higher and lower reporting levels, with intensive reporting activity in some years and fewer reports in others. In contrast, smaller IQR values were observed for diclofenac, celecoxib, and nimesulide (IQR 0.75), indicating more concentrated yearly counts. Regarding temporal distribution, the higher skewness value was observed for nimesulide (1.36), suggesting a right-skewed distribution of yearly reports, with a few years reporting many cases and several years reporting few cases. This pattern suggests uneven reporting over time due to the variability in reporting activity or external influences and should not be interpreted as a direct measure of time-constant risk (Supplementary Table 1). Given the small number of yearly observations (n = 10), shape indicators were interpreted descriptively.

**FIGURE 2:**
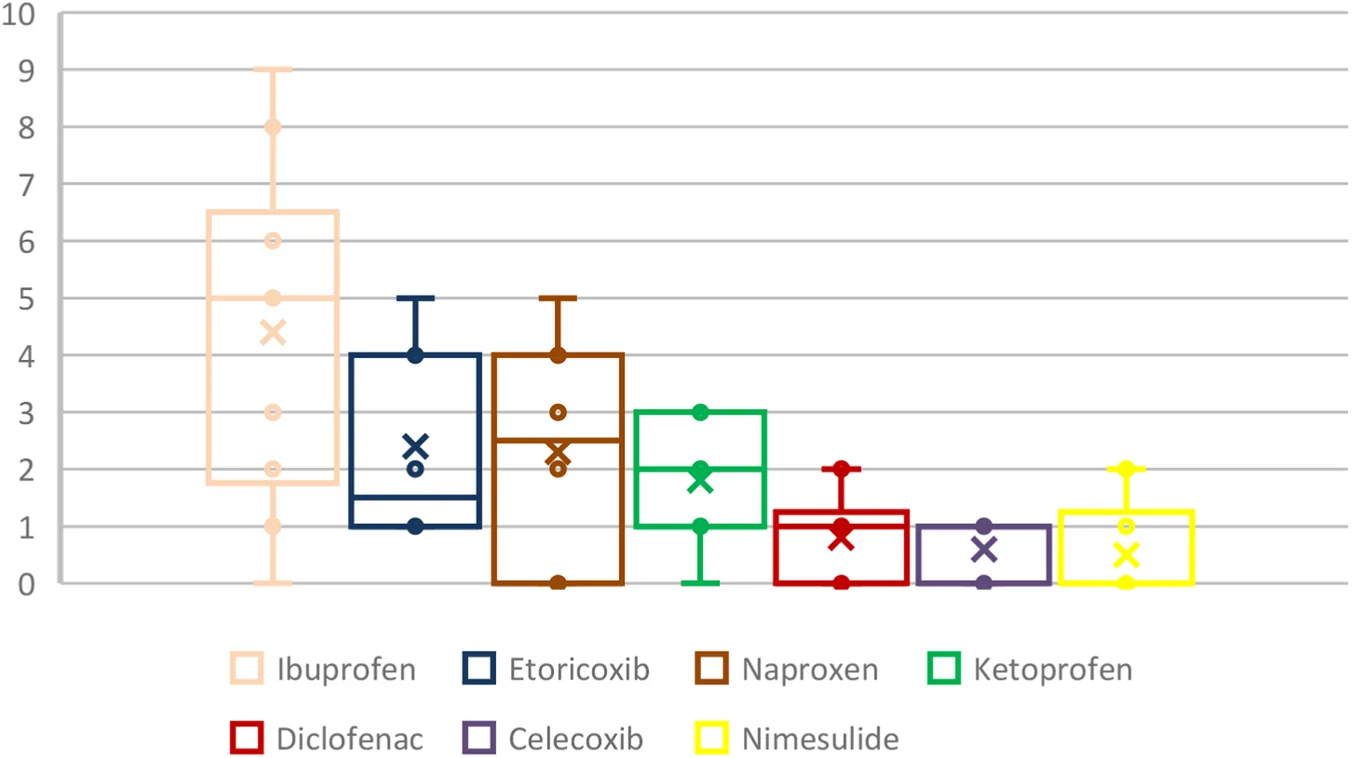
Distribution of ADRs reported for NSAIDs between 2015 and 2024. The box represents the interquartile range (Q1–Q3), the line inside the box represents the median, the markers indicate the mean, the inner points represent the real values, and the whiskers indicate the minimum and maximum values. No outliers were detected.

The distribution of ADRs for the seven NSAIDs studied was evaluated and visualized with boxplots (Figure 2). The median and IQR were calculated to assess central tendency and variability. Ibuprofen showed the highest variability, with a quartile 1 (Q1) of 2.25, Q3 of 5.75, and an IQR of 3.5, indicating a wide spread of ADRs reported. For diclofenac, celecoxib, and nimesulide, very low variability (IQRs: 1–1.25; medians at or near 0–1) was observed, indicating a uniform distribution of ADRs. Moreover, no outliers were detected for any of the drugs.

Supplementary Figure S1 presents the sex distribution of reported cases for all seven NSAIDs. Of the total cases (n = 128), 60.16% (n = 77) were reported among females and 36.72% (n = 47) among males. In only 4 cases (3.13%), the patient’s sex was not specified. ADR reports were more frequently observed in female patients than in male patients for each of the seven NSAIDs, with the highest proportion reported for nimesulide (80%) and naproxen (78.26%). The distribution of ADR reports for diclofenac was balanced between the two sexes (1:1), while for ibuprofen a slightly higher proportion of reports was observed in females (52.27%) compared with males (43.18%).

According to the data presented in Supplementary Figure S2, a high predominance was observed in the adult category (n = 83, 64.84% of ICSRs). A significant number of ICSRs (n = 23, 17.97%) were recorded in children and adolescents under 18 years, associated exclusively with ibuprofen. In older patients (65–84 years), the most frequently reported ADRs were associated with etoricoxib, ketoprofen, and naproxen, while for ibuprofen only a single ICSR was identified.

More than half of the ICSRs for the seven NSAIDs were submitted by non-healthcare professionals (n = 74). Physician-reported ICSRs accounted for 44 cases (34.38%), while pharmacist-reported ICSRs (n = 10) represented 7.81% of the total. This reporting pattern may be partly influenced by the legal status and accessibility of NSAIDs in Romania, as ibuprofen is available over the counter, whereas other NSAIDs require a prescription (Supplementary Figure S3).

The highest frequency of ADRs per report was observed with nimesulide (4.2 ADRs/report), and the lowest with diclofenac (1.88 ADRs/report). For the rest of the NSAIDs, a similar frequency was noticed (Supplementary Figure S4).

### 3.2 Analysis of reported adverse reactions for ibuprofen

**FIGURE 3:**
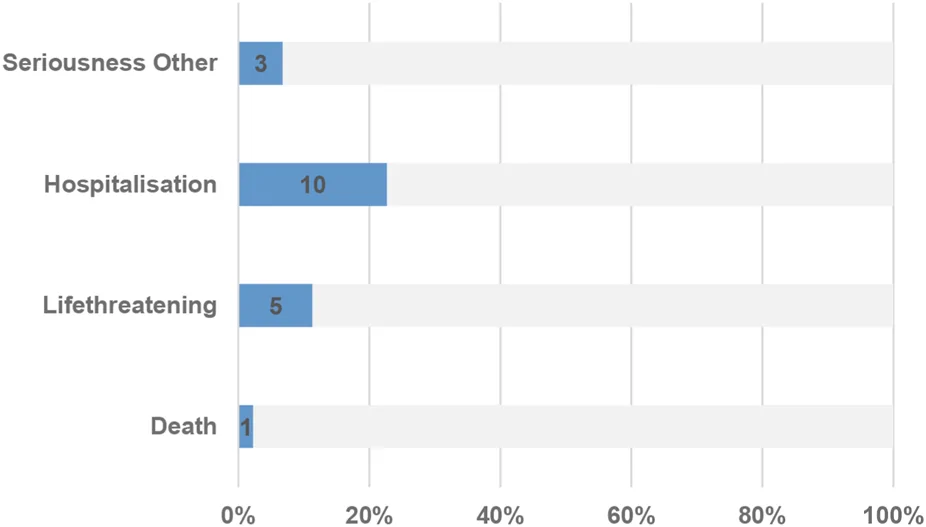
Distribution of serious events for ibuprofen by medical condition (n = 15 ICSRs). ICSR, individual safety case report.

**FIGURE 4:**
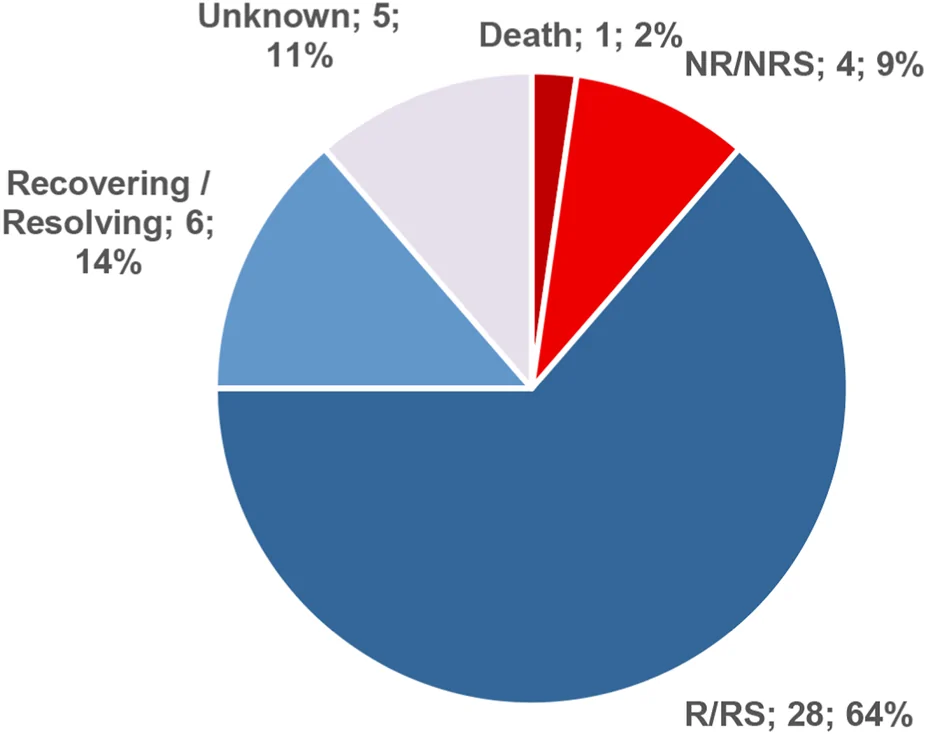
Distribution of cases according to the outcome (n = 44). NR/NRS, not recovered/not resolved, R/RS, recovered/resolved.

A total of 15 ICSRs were classified as serious. However, multiple seriousness criteria could be assigned to the same case; therefore, the cumulative number of seriousness criteria was 19 (Figure 3). The most frequently reported criteria were hospitalization or prolongation of hospitalization (n = 10), life-threatening condition (n = 5), other medically important condition (n = 3), and death (n = 1). As for the outcome, almost 80% of the cases had a favorable recovery/resolution (Figure 4). Because more than one criterion could apply to a single ICSR, categories were not mutually exclusive.

**FIGURE 5:**
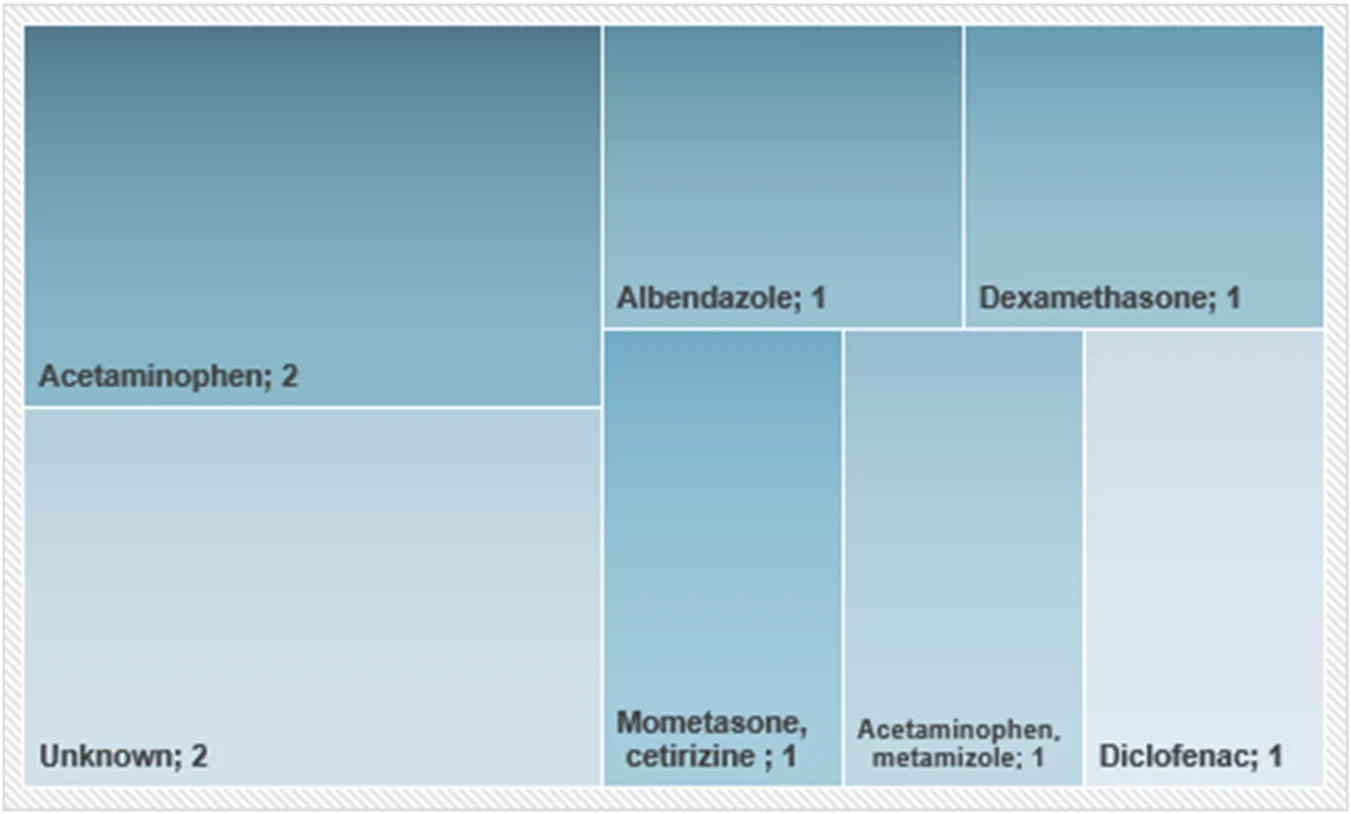
Distribution of cases in which ibuprofen is not the only suspect (n = 9).

Among the 44 ibuprofen-related ICSRs, 35 reports involved ibuprofen as the sole suspected drug, while 7 reports included co-reported medications. The most frequently reported co-medications were paracetamol (n = 2), albendazole (n = 1), dexamethasone (n = 1), diclofenac (n = 1), and combinations of mometasone with cetirizine (n = 1) and acetaminophen with metamizole (n = 1). In 2 ICSRs, concomitant medication information was not specified. Co-reported drugs were recorded as concomitant medications and were not assessed as additional suspected drugs (Figure 5).

**FIGURE 6:**
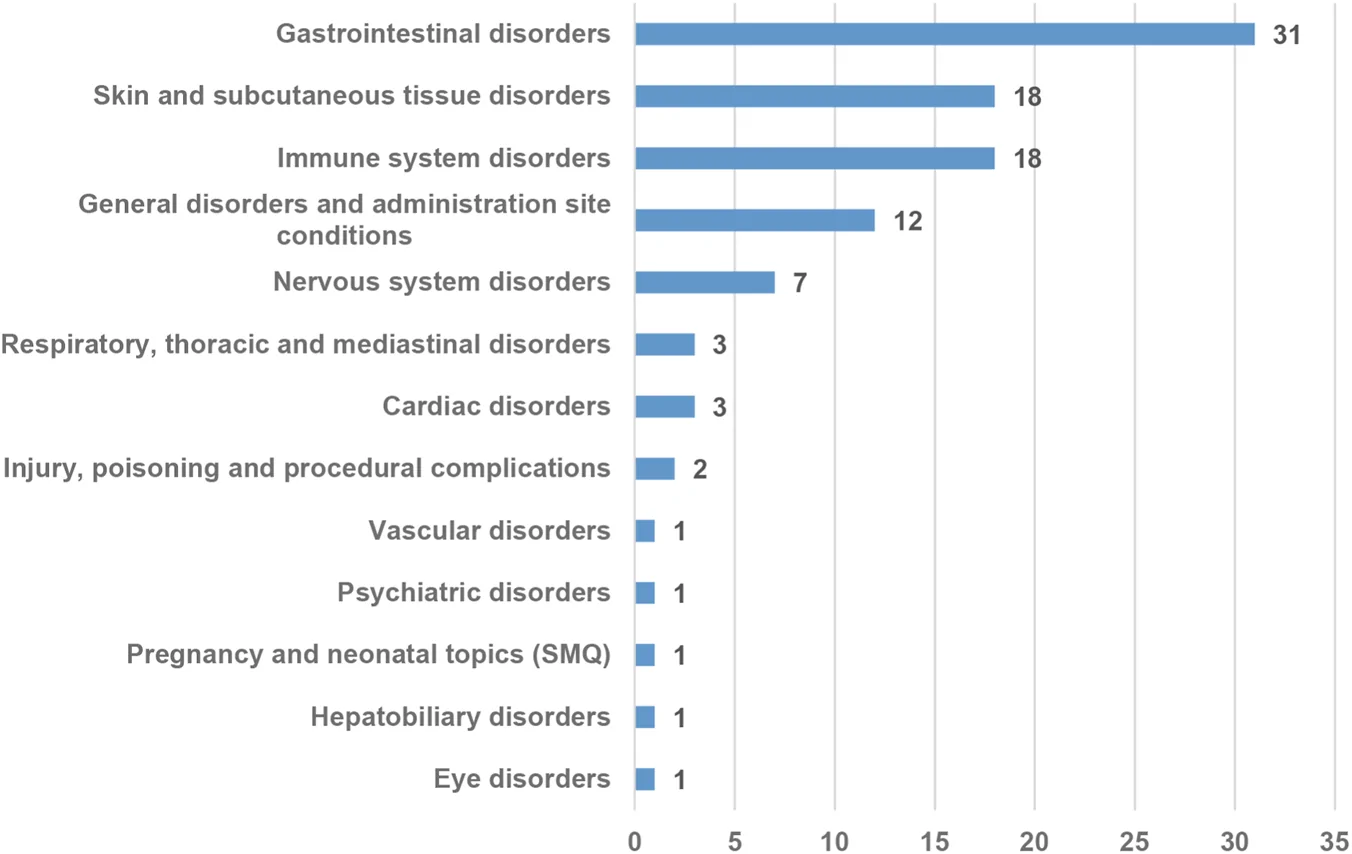
Distribution of reported ADRs by categories (n = 94).

A total of 44 ICSRs were submitted to the NAMMDR, containing 94 adverse drug reactions (ADRs), coded into 61 MedDRA Preferred Terms (PTs) across 13 SOCs. At the SOC level, the most frequently affected categories were Gastrointestinal disorders (n = 31 ADRs), Skin and subcutaneous tissue disorders (n = 18 ADRs), Immune system disorders (n = 18 ADRs), General disorders and administration site conditions (n = 12 ADRs), and Nervous system disorders (n = 7 ADRs) (Figure 6).

Gastrointestinal ADRs (n = 31) were predominantly represented by non-specific symptoms, including diarrhoea (n = 6), abdominal pain (n = 4), and nausea (n = 4) (Supplementary Figure S5a). However, clinically significant events were also observed within this SOC, including upper gastrointestinal hemorrhage, hematemesis, melena, and erosive hemorrhagic gastritis (each reported in individual cases).

Skin and subcutaneous tissue disorders (n = 18) included mainly hypersensitivity-related reactions, such as pruritus (n = 6), rash (n = 4), macular rash (n = 2), erythema, and urticaria (Supplementary Figure S5b).

Nervous system disorders (n = 7) were mainly represented by dizziness (n = 3), as well as other less frequently reported PTs including headache, somnolence, photophobia, and taste disturbance (Supplementary Figure S5c).

**FIGURE 7:**
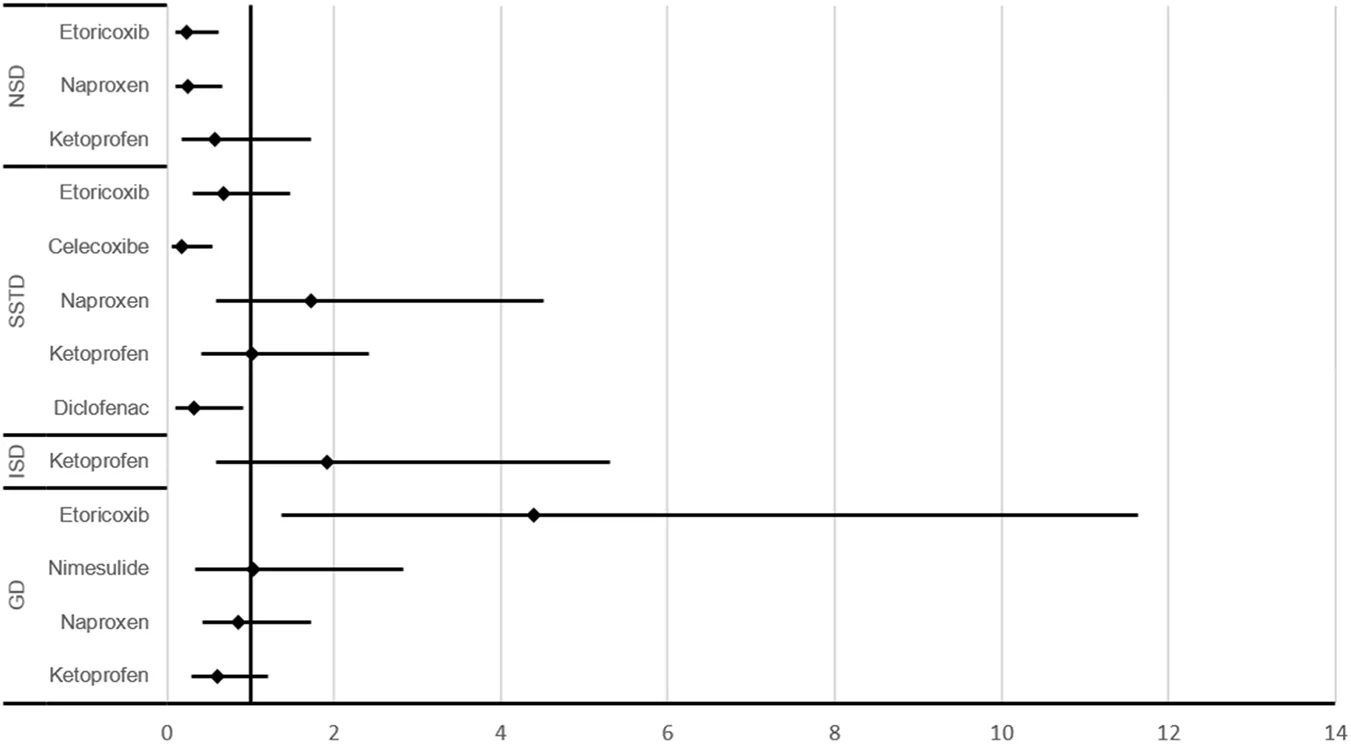
Disproportionality analysis for ibuprofen reports. GD, Gastrointestinal disorders; ISD, Immune system disorders; NSD, Nervous system disorders; SSTD, Skin and subcutaneous tissue disorders.

For the disproportionality analysis based on data submitted to the NAMMDR between 2015 and 2024, only System Organ Classes (SOCs) with more than five reports were included. Accordingly, the analysis was feasible for four SOCs and for selected comparator NSAIDs: Gastrointestinal disorders (ketoprofen, naproxen, nimesulide, and etoricoxib), Immune system disorders (ketoprofen), Nervous system disorders (ketoprofen, naproxen, and etoricoxib), and Skin and subcutaneous tissue disorders (diclofenac, ketoprofen, naproxen, and celecoxib) (Figure 7).

The results showed that Gastrointestinal disorders reports associated with ibuprofen exhibited higher disproportional reporting compared with etoricoxib (ROR: 4.39; 95% CI: 1.66–11.63). In contrast, lower disproportional reporting was observed for Skin and subcutaneous tissue disorders and Nervous system disorders when comparing ibuprofen with diclofenac (ROR: 0.32; 95% CI: 0.11–0.92), celecoxib (ROR: 0.17; 95% CI: 0.06–0.54), naproxen (ROR: 0.26; 95% CI: 0.10–0.67), and etoricoxib (ROR: 0.24; 95% CI: 0.09–0.63) (Supplementary Table 2).

For the remaining SOCs and comparisons, no statistically significant disproportionality reportingwas observed based on the available NAMMDR data.

## 4 Discussion

This study provides a long-term analysis of ibuprofen-associated adverse reactions (ADRs) reported by healthcare professionals and patients to the national database of the NAMMDR over a 10-year period (between 2015 and 2024) and compares the reporting profile of ibuprofen with that of six other NSAIDs. The disproportionality analysis identified a higher reporting frequency of Gastrointestinal disorders for ibuprofen compared with etoricoxib. Skin and subcutaneous tissue disorders and Nervous system disorders were reported less disproportionately than for several comparator NSAIDs. These findings are consistent with the established pharmacological profile of ibuprofen and the known gastrointestinal safety concerns associated with non-selective COX inhibition. Importantly, the observed reporting patterns were derived from real-world national pharmacovigilance data and provide information on the local safety profile of ibuprofen in routine clinical practice. The analysis did not include the ADRs submitted by Marketing Authorization Holders (MAH) to the EV database.

During the analyzed period, a relatively small number of reports (n = 128) were submitted for the 7 anti-inflammatory drugs under analysis, of which ibuprofen showed the most reports (n = 44), accounting for 94 ADRs. The higher number of reports for ibuprofen may reflect its substantially greater population exposure, due to widespread availability in both prescription and over-the-counter formulations in Romania and worldwide (Austin et al., 2022; Green et al., 2026), which may increase reporting frequency independently of intrinsic toxicity, ibuprofen is considered a relatively safe medicine (Varrassi et al., 2020; Rainsford, 2009).

A highly variable number of reports was observed annually, with a decline in reporting during 2020–2022; this fluctuation may be attributed to the COVID-19 pandemic, as only six reports were recorded during that interval, and the population focused on the newly approved vaccines, a high number of ICSRs related to these medicines being recorded in EV (Tobaiqy et al., 2021; Abbattista et al., 2021).

A predominance of female reports was observed for most of the evaluated drugs; however, diclofenac and ibuprofen demonstrated a comparatively balanced sex distribution in reported cases. These findings may reflect differences in drug exposure, prescription practices, or reporting behavior rather than true sex-based differences in ibuprofen behavior (Austin et al., 2022; Khalil et al., 2019; Paixão et al., 2018).

An additional finding of interest was the predominance of reports involving children and adolescents, who accounted for more than half of all ibuprofen-related reports. Although this observation may partly reflect greater exposure and use of ibuprofen in pediatric populations, it emphasizes the importance of continued pharmacovigilance monitoring in younger patients.52% of ibuprofen cases were reported in the pediatric population (<18 years). The high percentage may be influenced by two components: first, ibuprofen is frequently prescribed in this age group due to its anti-inflammatory and antipyretic effects (de Martino et al., 2017), second - accessible reporting systems (Vassallo et al., 2025) on the other hand. Pediatric groups from other European countries, like Italy, have also reported ADRs to ibuprofen to their national database (Pelliccia et al., 2021). In Germany, for the pediatric population, ibuprofen was reported to be the second most suspected drug related to reports submitted for a period of 20 years, starting with 2000, generating on average 24 reports per year (Dubrall et al., 2021).

Individuals aged 65 and over accounted for the largest proportion of reports for etoricoxib, ketoprofen, and naproxen. This pattern likely reflects higher exposure in older patients, given the increased prevalence of chronic inflammation and degenerative conditions in this age group (Khalil et al., 2019; Aguiñaga-Martínez et al., 2024) although age associated pharmacokinetic changes and increased comorbidity burden could also influence adverse event reporting (Ngcobo, 2025; Tang and Chan, 2012).

Approximately 60% of the reports originated from patients, while the rest were submitted by healthcare professionals, the majority of whom were doctors, and a small number by pharmacists, who can recommend only ibuprofen; the other six anti-inflammatory agents being prescription drugs. In general, patient reports tend to emphasize subjective symptoms, whereas healthcare professionals typically provide more objective clinical documentation (Rolfes et al., 2015). A similar behavior was reported by a large-scale study, analyzing several countries from Europe, Asia, America, and Africa (Worakunphanich et al., 2022). The importance of providing patients with the possibility of direct reporting is recognized by the scientific community (Sienkiewicz et al., 2021) and several methods to disseminate the information to the public have been practiced by the authorities (Matos et al., 2016), triggering an increased awareness from the public, proportional to the growth in the number of reports submitted by the general population (Valinciute-Jankauskiene and Kubiliene, 2021).

For ibuprofen, in the majority of analyzed cases, the outcome was favorable (80%), although some required hospitalization or prolonged hospital stay. This finding aligns with the documented safety profile of ibuprofen, as stated by the Summary of Product Characteristics, where both mild and severe side effects are mentioned (Electronic Medicines Compendium (emc), 2026), but overall, ibuprofen is considered safe, including for infants from 3 months old and 5 kg or more bodyweight (Ziesenitz et al., 2022).

Overall, the 44 reports submitted to NAMMDR for ibuprofen included 94 ADRs described using 61 Preferred Terms across 13 System Organ Classes (SOCs). The most frequently reported SOCs were Gastrointestinal disorders (32%), Skin and subcutaneous tissue disorders (19%), Immune system disorders (19%), General disorders and administration site conditions (12%), and Nervous system disorders (7.5%).

Most ADRs identified in our study involved the Gastrointestinal disorders (32%), the most reported being diarrhoea, abdominal pain, and nausea. All three ADRs are mentioned in the Summary of Product Characteristics (SmPC) (Electronic Medicines Compendium (emc), 2026). Through its mechanism of action, ibuprofen non-selectively inhibits cyclooxygenase (COX)-1 and COX-2 enzymes, leading to alterations in gastric mucosal protective mechanisms (Rainsford, 2009). Diarrhoea should quickly become a concern in the case of infants, because it can lead to dehydration (Barbagallo and Sacerdote, 2019).

Cutaneous adverse reactions also represented a significant proportion of the recorded events, including pruritus and rash. Inhibition of COX-1 may divert arachidonic acid metabolism towards the 5-lipoxygenase pathway, resulting in increased production of cysteinyl leukotrienes, which are implicated in the cutaneous manifestations associated with non-selective nonsteroidal anti-inflammatory drugs (NSAIDs) (Szczeklik 1990). Among Immune system disorders, anaphylactic shock—a serious reaction—was reported in two cases. The literature describes anaphylactic shock associated with ibuprofen as a rare event (Shaikhain et al., 2019).

Most of the frequently reported ADRs identified in this study, including diarrhoea, abdominal pain, nausea, pruritus, rash, urticaria, and anaphylactic reactions, are already described in the SmPC of ibuprofen (Electronic Medicines Compendium (emc), 2026). Therefore, the present analysis did not identify previously unrecognized ADRs. However, it contributes additional evidence regarding the relative reporting patterns of these reactions within the Romanian population and highlights gastrointestinal events as the most prominent category of ADRs associated with ibuprofen.

In this retrospective analysis, disproportionality analysis was conducted only for SOCs comprising more than five reports. Consequently, only four SOCs and a subset of the comparator medicinal products (other NSAIDs) were eligible for evaluation.

Regarding the reporting of Nervous system disorders to NAMMDR, ibuprofen showed a lower disproportional reporting than etoricoxib (ROR, 95% CI) and naproxen. According to the results of our study, Gastrointestinal disorders associated with ibuprofen were reported with a higher disproportional reporting compared to etoricoxib, a selective COX-2 inhibitor. A possible explanation may lie in the non-selective inhibition of both COX-1 and COX-2 by ibuprofen, which may compromise gastric mucosal protective mechanisms (Takemoto et al., 2008).

The findings of the present national analysis of ibuprofen are broadly consistent with safety information reported in international pharmacovigilance databases, such as FAERS and EudraVigilance (Buciuman et al., 2026; Buciuman et al., 2025a; Buciuman et al., 2025b).

### 4.1 Limitations and perspectives

The results of the study should be interpreted considering the inherent limitations of pharmacovigilance databases, among which the lack of denominators, underreporting, the spontaneous nature of the reports, missing information in some reporting criteria, and the small-number effect should be considered (Vonica et al., 2024 and Dobrea et al., 2024). An important limitation of the study derives from the small ratio of reports versus the broad use profile of ibuprofen, which is a well-known drug, available even over the counter, variant that has gained its status precisely because of the lack of serious side effects and also because of its long-term use. Increased accessibility and frequent over-the-counter use may lead to underreporting of adverse reactions, especially in the context of pharmacovigilance systems predominantly based on spontaneous reporting. Additional variability may also arise from differences in prescribing patterns and formulations between ibuprofen and the comparator NSAIDs, particularly given that ibuprofen is widely available over the counter whereas several comparators are prescription-only medicines. Another limitation of the study is represented by the current context of pharmacovigilance activities in Romania. The reporting of adverse drug reactions is mostly done spontaneously, being influenced by the varying level of information and involvement of health professionals. In practice, the reporting rate seems to be mainly determined by existing legal obligations, as well as by the level of professional training previously acquired, with indications that older generations of doctors have benefited from more consistent training in the field of pharmacovigilance (Paveliu et al., 2013). This context may contribute to underreporting and possible distortion of the data analyzed, thus influencing the accuracy of the estimation of the safety profile assessed in the study.

Although a direct causal relationship between the suspected ADR and the drug cannot be established, disproportionality analysis methods are endorsed and used by the European Medicines Agency and other regulatory organisms for pharmacovigilance signal detection European Medicine Agency (2016), even in small databases (Caster et al., 2020).

A disproportionality analysis was conducted to identify reporting trends and raise clinicians’ awareness of targeted investigations in these specified directions. The identified adverse drug reaction reporting patterns may support clinicians in recognizing potential safety concerns associated with ibuprofen, particularly gastrointestinal, cutaneous, and hypersensitivity reactions, especially in patients with relevant risk factors or comorbidities. These findings underscore the importance of individualized risk–benefit assessment and appropriate clinical monitoring when prescribing ibuprofen, in line with current clinical guidelines and established safety recommendations. Given the small number of reports and the resulting sparse data across several 2 × 2 contingency tables, the resulting ROR estimates may be unstable, with wide confidence intervals reflecting statistical uncertainty. Therefore, the findings should be interpreted as exploratory disproportionality patterns.

## 5 Conclusion

A retrospective analysis of adverse reaction reports submitted to the NAMMDR national database between 2015 and 2024 for ibuprofen and six other anti-inflammatory drugs was conducted. The results highlighted a fluctuating trend in reporting over the years, with a higher frequency among the pediatric population, for ibuprofen, respectively among the older population for etoricoxib, ketoprofen and naproxen. Also, the safety profile of ibuprofen, as reflected in the NAMMDR database, showed a higher proportion of reported adverse reactions within the Gastrointestinal disorders SOC compared with Immune system disorders, Skin and subcutaneous tissue disorders, or Nervous system disorders SOCs. The comparison in the proposed disproportionality analysis with molecules from the same therapeutic area was limited by the relatively small number of reports recorded, with Gastrointestinal disorder reports involving ibuprofen showing higher disproportional reporting compared with etoricoxib. No unexpected ADR patterns or previously undescribed safety concerns were identified; however, the study provides new national-level evidence regarding the comparative reporting profile of ibuprofen relative to other NSAIDs and identifies pediatric patients as the most frequently represented reporting population in Romania. The findings of this study should be interpreted within the methodological framework of spontaneous reporting systems, where underreporting of adverse drug reactions and variability of existing data are recognized limitations. Patient-reported data may introduce heterogeneity in clinical detail; however such reports represent a valuable component of real-world pharmacovigilance.

## Data Availability

The original contributions presented in the study are included in the article/Supplementary Material, further inquiries can be directed to the corresponding authors.
