# Supplementary material for: Safety assessment for ibuprofen using disproportionality analysis: a decade of data from the Romanian national pharmacovigilance system
Source: Front Pharmacol. 2026 Aug 12;17:1863824. doi: 10.3389/fphar.2026.1863824 (PMC13506849; doi:10.3389/fphar.2026.1863824)
Supplement: Supplementary file 1 [file Supplementaryfile1.docx]

**Supplementary Material**

Table 1. Descriptive statistical analysis of ADR reports for selected NSAIDs (2015–2024).

|  | Ibuprofen | Diclofenac | Ketoprofen | Naproxen | Nimesulide | Celecoxib | Etoricoxib |
| --- | --- | --- | --- | --- | --- | --- | --- |
| Total number of reports | 44 | 9 | 18 | 23 | 5 | 7 | 24 |
| Proportion of ADRs | 34.1% | 6.2% | 14.0% | 17.8% | 3.9% | 5.4% | 18.6% |
| Mean | 4.89 | 1.33 | 2.00 | 3.29 | 1.67 | 1.17 | 2.40 |
| Standard Error | 0.87 | 0.21 | 0.29 | 0.42 | 0.33 | 0.17 | 0.52 |
| Median | 5 | 1 | 2 | 3 | 2 | 1 | 1.5 |
| Mode | 5 | 1 | 1 | 2 | 2 | 1 | 1 |
| Standard Deviation | 2.62 | 0.52 | 0.87 | 1.11 | 0.58 | 0.41 | 1.65 |
| Sample Variance | 6.86 | 0.27 | 0.75 | 1.24 | 0.33 | 0.17 | 2.71 |
| Kurtosis | -0.63 | -1.88 | -1.71 | -0.94 | NA | 6.00 | -1.83 |
| Skewness | 0.10 | 0.97 | 0.00 | 0.25 | -1.73 | 2.45 | 0.50 |
| Range | 8 | 1 | 2 | 3 | 1 | 1 | 4 |
| Minimum | 1 | 1 | 1 | 2 | 1 | 1 | 1 |
| Maximum | 9 | 2 | 3 | 5 | 2 | 2 | 5 |
| Count (years with reports) | 9 | 6 | 9 | 7 | 3 | 6 | 10 |
| Coefficient of Variation | 53.48% | 38.34% | 43.00% | 33.84% | 34.33% | 34.48% | 68.33% |
| Outliers | None | None | None | None | None | 2 (2010) | None |

Table 2. Table X. 2 × 2 contingency tables used for reporting odds ratio (ROR) calculation by system organ class (SOC). GD - Gastrointestinal disorders; ISD - Immune system disorders; NSD - Nervous system disorders; SSTD - Skin and subcutaneous tissue disorders.

| SOC level | Ibuprofen: ADRs reported in SOC of interest  (a) | Ibuprofen: ADRs reported in other SOCs  (b) | Comparator drug | Comparator: ADRs reported in SOC of interest  (c) | Comparator: ADRs reported in other SOCs  (d) | ROR | 95% CI lower bond | 95% CI upper bond |
| --- | --- | --- | --- | --- | --- | --- | --- | --- |
| GD | 31.5 | 68.5 | Ketoprofen | 22.5 | 29.5 | 0.60 | 0.30 | 1.20 |
| GD | 31.5 | 68.5 | Naproxen | 19.5 | 36.5 | 0.86 | 0.43 | 1.72 |
| GD | 31.5 | 68.5 | Nimesulide | 6.5 | 14.5 | 1.03 | 0.37 | 2.84 |
| GD | 31.5 | 68.5 | Etoricoxib | 5.5 | 52.5 | 4.39 | 1.66 | 11.63 |
| ISD | 18.5 | 81.5 | Ketoprofen | 5.5 | 46.5 | 1.92 | 0.69 | 5.31 |
| SSTD | 18.5 | 81.5 | Diclofenac | 7.5 | 10.5 | 0.32 | 0.11 | 0.92 |
| SSTD | 18.5 | 81.5 | Ketoprofen | 9.5 | 42.5 | 1.02 | 0.43 | 2.41 |
| SSTD | 18.5 | 81.5 | Naproxen | 6.5 | 49.5 | 1.73 | 0.66 | 4.52 |
| SSTD | 18.5 | 81.5 | Celecoxibe | 8.5 | 6.5 | 0.17 | 0.06 | 0.54 |
| SSTD | 18.5 | 81.5 | Etoricoxib | 14.6 | 43.5 | 0.68 | 0.31 | 1.47 |
| NSD | 7.5 | 92.5 | Ketoprofen | 6.5 | 45.5 | 0.57 | 0.19 | 1.72 |
| NSD | 7.5 | 92.5 | Naproxen | 13.5 | 42.5 | 0.26 | 0.10 | 0.67 |
| NSD | 7.5 | 92.5 | Etoricoxib | 14.6 | 43.5 | 0.24 | 0.09 | 0.63 |

a, b, c, d correspond to the cells of the 2 × 2 contingency tables used for ROR calculation.

**Figure 1.** Distribution of cases by sex (n=128). NS – not specified

**Figure 2.** Distribution of cases by age category (n=128). NS – not specified

**Figure 3.** Distribution of cases by reporter category (n=128). Non-HP – non-healthcare professional.

**Figure 4.** The comparison between the number of ADRs/report

a

b

c

d

e

**Figure 5.** The distribution of the most common ADRs by category: (a) – gastrointestinal, (b) dermal, (c) – neurological, (d) – immunological and (e) – general.
